# Difference in Clinical Features between Right- and Left-Sided Acute Colonic Diverticulitis

**DOI:** 10.1038/s41598-020-60397-5

**Published:** 2020-02-28

**Authors:** Kil-yong Lee, Jaeim Lee, Youn Young Park, Younglim Kim, Seong Taek Oh

**Affiliations:** 1 Division of Coloproctology, Department of Surgery, Uijeongbu St. Mary’s Hospital, College of Medicine, The Catholic University of Korea, Uijeongbu-si, South Korea; 2 Department of Surgery, Seoul Asan Medical Center, Ulsan University College of Medicine, Seoul, Korea

**Keywords:** Gastroenteritis, Colitis

## Abstract

Right colonic diverticulitis (RCD) and left colonic diverticulitis (LCD) may have different clinical features due to the different embryologic origins and anatomical locations of each colon. Therefore, we aimed to compare RCD and LCD in terms of the associated clinical features. We retrospectively collected clinical data from patients who were diagnosed with acute colonic diverticulitis based on computed tomography findings between 2011 and 2017. RCD was defined as colonic diverticulitis extending from the caecum to the transverse colon, and LCD was defined as extending from the splenic flexure to the sigmoid colon. These analyses included 667 patients with RCD and 58 patients with LCD. Relative to the patients with LCD, the patients with RCD were younger (P < 0.001), were more likely to be male (P = 0.033), were taller (P < 0.001), had lower body mass index values (P < 0.001), had less advanced modified Hinchey stages (P < 0.001), and had shorter hospital stays (P < 0.001). Having LCD rather than RCD was a predictor of recurrent colonic diverticulitis (P = 0.003). Relative to LCD, RCD occurs at younger ages, is expressed at less advanced modified Hinchey stages, and is associated with lower risks of recurrence.

## Introduction

The clinical importance of colonic diverticulitis has been emphasised in the literature because the incidence of colonic diverticulitis has been increasing worldwide^1^. Several studies have been conducted to evaluate left colonic diverticulitis (LCD) and identify the best treatment strategy because it is common in Western populations^2,3^. In contrast, right colonic diverticulitis (RCD) is rare in Western populations but common in Asian populations^4,5^. Despite its prevalence in Asian populations, few studies have investigated the clinical course of RCD or sought to identify the best treatment strategy. Recently, Strate *et al*.^6^ introduced a management algorithm for colonic diverticulitis that was mainly based on LCD. As a result, the algorithm has limited applicability to patients with RCD. For example, the algorithm recommends that only patients with complicated diverticulitis undergo surgery. It further recommends that if surgery is performed, then second-stage surgery should also be performed depending on the patient’s hemodynamic stability. However, the medical literature contains few cases of RCD in Asian patients for whom complicated diverticulitis was an indication for surgery^7^.

The left and right colons have different embryologic origins, with the left colon originating from the hindgut and the right colon originating from the midgut^8^. Right diverticular disease and left diverticular disease differ in that diverticula from the left colon are more likely to be true diverticula^8^. Therefore, the clinical characteristics of LCD and RCD may differ. Thus far, a few studies have compared RCD and LCD, but these studies have been severely limited by their small sample sizes^9,10^. We therefore aimed to perform a similar study with a larger sample size.

## Methods

This study was approved by the institutional review board (IRB) of the Catholic University of Korea and was performed in accordance with the IRB’s guidelines and regulations. Informed consent was waived under IRB approval.

### Patients

For these analyses, we considered all patients who were diagnosed with acute colonic diverticulitis based on computed tomography (CT) findings at our hospital between January 2011 and December 2017. Whenever a patient was diagnosed with acute colonic diverticulitis, whether at an outpatient clinic or in the emergency room, hospitalisation was recommended, but patients who did not consent to hospitalisation were discharged with oral antibiotics. We excluded patients who had both RCD and LCD and patients who were initially diagnosed with diverticulitis but whose diagnoses were corrected to colon cancer based on follow-up colonoscopy findings. Clinical data were retrospectively collected from electronic medical records.

### Variables

The collected data included physical information such as age, sex, height, body mass index (BMI), body temperature, histories of smoking and alcohol consumption, medical histories (e.g. histories of hypertension, diabetes, and cardiac disease), modified Hinchey stages, laboratory findings (e.g. white blood cell counts and C-reactive protein levels), hospital stay durations, and the occurrence of recurrent colonic diverticulitis.

### Definitions

Acute colonic diverticulitis was diagnosed based on the CT findings at the initial visit. The findings of interest were localised thickening of the colonic wall to ≥5 mm and signs of inflammation of the pericolic fat. The presence or absence of abscess formation, extraluminal air, and extraluminal contrast was not considered diagnostically relevant^11^. RCD was defined as colonic diverticulitis that extended from the caecum to the transverse colon, and LCD was defined as colonic diverticulitis that extended from the splenic flexure to the sigmoid colon. The follow-up period was defined as extending from the first diagnosis to the last follow-up assessment.

### Follow-up protocol

The patients were discharged following the resolution of their clinical symptoms and leucocytosis. We recommended that they undergo colonoscopies 4–8 weeks after discharge and that they visit the hospital for follow-up assessments every 6 months to monitor for recurrence. We also recommended that they visit our hospital if they experienced recurrent abdominal pain. CT imaging was used to assess cases of suspected recurrent colonic diverticulitis.

### Statistical analyses

Between-group comparisons of continuous variables were performed with Student’s t-test and the Chi-square test, and between-group comparisons of categorical variables were performed with Fisher’s exact test and the linear-by-linear association test. Kaplan-Meier analysis with the log-rank test was used to compare the RCD and LCD groups in terms of recurrence rates. Cox proportional regression analysis was used for a preliminary identification of the predictors of recurrence, and predictors that had 2-sided P values <0.20 were included in a multivariable analysis involving a backward-elimination approach.

## Results

**Figure 1:**
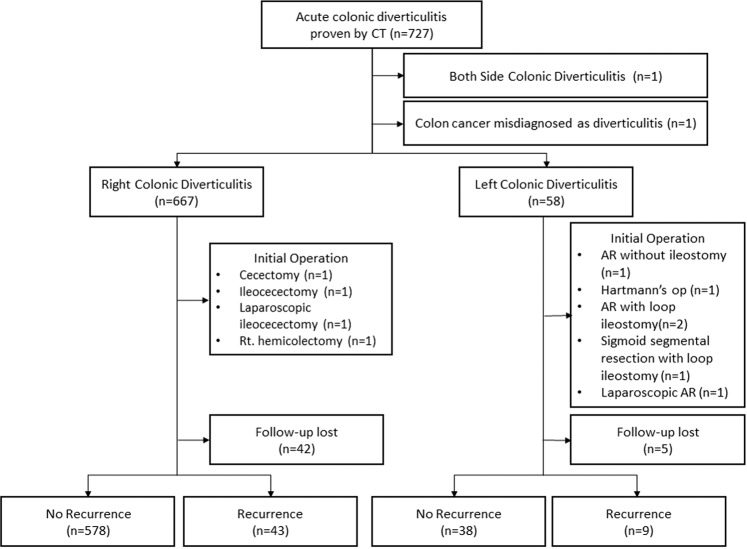
Patient selection flow chart.

We identified 727 patients who met our initial search criteria. After we excluded 1 patient who had bilateral inflammation and 1 patient who was found to have colon cancer rather than colonic diverticulitis, our study sample included 725 consecutive patients. Of these patients, 667 had RCD, and 58 had LCD. Emergency surgery was performed in 4 patients with RCD (0.6%) and in 6 patients with LCD (10.3%) (Fig. 1).

Most of the patients were referred to our hospital from other clinics, but the underlying reason for referral was generally not a lack of clinical improvement. In fact, only 2 patients, both of whom had RCD, were referred for that reason. One of those patients had been treated with intravenous antibiotics for 9 days, and the other had been treated with intravenous antibiotics for 12 days.

The clinical characteristics of the RCD and LCD groups are shown in Table 1. Relative to the patients with LCD, the patients with RCD were younger (P < 0.001), were more likely to be male (P = 0.033), were taller (P < 0.001), and had lower BMIs (P < 0.001). However, the patients with LCD had more advanced modified Hinchey stages (P < 0.001) and longer hospital stays (P < 0.001). Furthermore, the RCD and LCD groups differed in terms of percentages of Westerners and Asians (P < 0.001), with the RCD group being mostly composed of Asians (665/667; 99.7%) and the LCD group including 5 Westerners (8.6%) and 53 Asians (91.4%). In-hospital mortality occurred for 3 patients with RCD and 2 patients with LCD. The causes of death were sepsis after emergency surgery (1 patient each in both groups) and myocardial infarction (2 patients in the RCD group and 1 patient in the LCD group).

**Table 1:** Comparison of the RCD and LCD groups.

	RCD group (n = 667)	LCD group (n = 58)	P Value
Age (y)	41.4 ± 12.8	58.8 ± 15.3	**<0.001**
Sex			**0.033**
Male	395 (59.2%)	26 (44.8%)	
Female	272 (40.8%)	32 (55.2%)	
Height (cm)	166.8 ± 8.7	161.4 ± 10.6	**<0.001**
BMI (kg/m^2^)	24.1 ± 3.5	26.1 ± 4.0	**<0.001**
Prehospital duration of symptoms (d)	2.1 ± 2.7	5.8 ± 20.0	0.160
Body temperature (°C)	36.7 ± 0.6	36.8 ± 0.7	0.294
Smoking	298 (44.7%)	12 (20.7%)	**<0.001**
Alcohol	261 (40.8%)	18 (31.0%)	0.144
Underlying disease			
Hypertension	88 (13.2%)	22 (37.9%)	**<0.001**
Diabetes	29 (4.4%)	8 (13.8%)	**0.002**
Cardiac disease	16 (2.4%)	4 (6.9%)	0.068
History of aspirin or NSAID usage	20 (3%)	5 (8.6%)	**0.024**
Modified Hinchey stage			**<0.001**
0	3 (0.4%)	1 (1.7%)	
Ia	618 (92.7%)	31 (53.4%)	
Ib	41 (6.1%)	13 (22.4%)	
II	4 (0.6%)	5 (8.6%)	
III	1 (0.1%)	4 (6.9%)	
IV	0 (0%)	4 (6.9%)	
Laboratory findings			
WBC (10^3^/μL)	11.6 ± 3.4	10.9 ± 3.8	0.168
Segment neutrophil (%)	71.5 ± 8.9	72.8 ± 10.9	0.389
CRP (mg/dL)	5.0 ± 4.7	7.4 ± 7.0	**0.013**
Hospital stay (d)	4.4 ± 2.3	6.7 ± 4.3	**<0.001**

Abbreviations: BMI, body mass index; CRP, C-reactive protein; LCD, left colonic diverticulitis; NSAID, nonsteroidal anti-inflammatory drug; RCD, right colonic diverticulitis; WBC, white blood cell.

**Figure 2:**
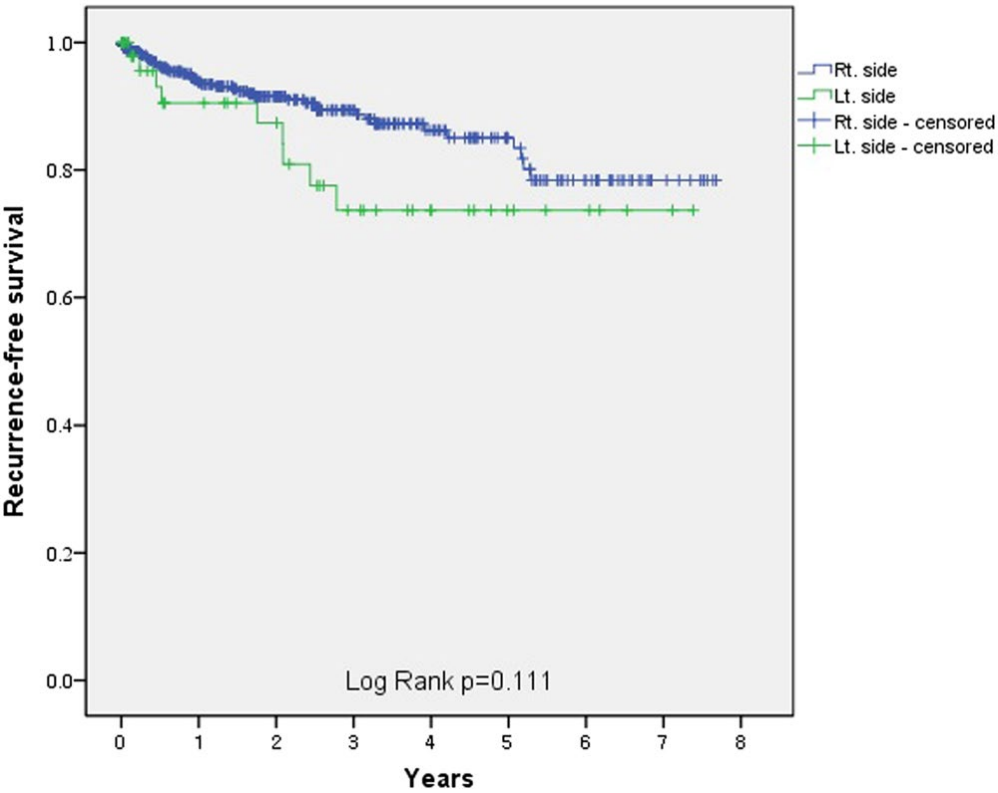
Kaplan-Meier curves for recurrence in the RCD group (blue line) and the LCD group (green line). Abbreviations: LCD, left colonic diverticulitis; RCD, right colonic diverticulitis.

The mean follow-up duration was 641.6 days (interquartile range, 44–1094.3 days), and 47 patients were lost to follow-up. Follow-up colonoscopy assessments were performed for 382 patients. Follow-up colonoscopies were performed for 351 patients in the RCD group (52.6%), and adenomas were detected in 88 of them (25.1%). Follow-up colonoscopies were performed for 31 patients in the LCD group (53.4%), and adenomas were detected in 8 of them (25.8%). During follow-up, the overall recurrence rate was 7.8% (n = 52), and the group-specific recurrence rates for the RCD and LCD groups were 6.9% (n = 43) and 19.1% (n = 9), respectively (Fig. 1). The log-rank test showed that this was not a significant between-group difference (P = 0.111) (Fig. 2). A multivariable analysis showed that the predictors of recurrence were younger ages (P = 0.026) and having LCD rather than RCD (P = 0.003) (Table 2).

**Table 2:** Risk factors for recurrence of colonic diverticulitis.

	Univariable analysis		Multivariable analysis	
	HR (95% CI)	P Value	HR (95% CI)	P Value
Age (y)	0.99 (0.97–1.01)	0.417	0.98 (0.96–1.00)	**0.026**
Sex^†^	0.85 (0.49–1.49)	0.574		
Height (cm)	1.02 (0.99–1.05)	0.330		
BMI	0.99 (0.91–1.07)	0.755		
Prehospital symptom duration	1.00 (0.98–1.03)	0.997		
Body temperature (°C)	0.88 (0.52–1.47)	0.614		
Initial pain score	0.93 (0.77–1.12)	0.440	0.87 (0.70–1.08)	0.200
Smoking	1.41 (0.81–2.44)	0.223	1.50 (0.84–2.66)	0.169
Alcohol	1.07 (0.61–1.90)	0.812		
Hypertension	1.21 (0.62–2.36)	0.573		
Diabetes	1.96 (0.84–4.61)	0.121		
Cardiac disease	0.60 (0.08–4.37)	0.616		
WBC	1.01 (0.93–1.09)	0.882		
Segment neutrophil (%)	0.99 (0.97–1.02)	0.677		
CRP	1.00 (0.95–1.06)	0.931		
Location of diverticulitis^‡^	1.78 (0.87–3.66)	0.116	3.46 (1.51–7.90)	**0.003**

^†^reference condition: male, ^‡^reference condition: right side.

Abbreviations: BMI, body mass index; CI, confidence interval; CRP, C-reactive protein; HR, hazard ratio; WBC, white blood cell.

Because the majority of the patients in our study had RCD, we performed a subgroup analysis of the predictors of recurrent RCD. In the multivariable analysis, longer prehospital symptom durations predicted recurrent RCD (P = 0.039), and younger ages were near-significant predictors of recurrent RCD (P = 0.075) (Table 3).

**Table 3:** Risk factors for recurrent RCD.

	Univariable analysis		Multivariable analysis	
	HR (95% CI)	P Value	HR (95% CI)	P Value
Age (y)	0.99 (0.97–1.01)	0.330	0.98 (0.96–1.00)	0.075
Sex^†^	0.73 (0.39–1.36)	0.320		
Height (cm)	1.03 (0.99–1.06)	0.158		
BMI	0.95 (0.87–1.04)	0.241		
Prehospital symptom duration	1.08 (1.01–1.15)	**0.033**	1.08 (1.00–1.16)	**0.039**
Body temperature (°C)	0.79 (0.44–1.43)	0.442		
Initial pain score	0.90 (0.73–1.12)	0.342		
Smoking	1.80 (0.98–3.31)	0.058	1.66 (0.89–3.12)	0.111
Alcohol	1.18 (0.63–2.19)	0.608		
Hypertension	1.12 (0.50–2.51)	0.789		
Diabetes	2.31 (0.83–6.48)	0.111		
Cardiac disease	0.05 (0.00–171.66)	0.466		
WBC	1.02 (0.93–1.11)	0.710		
Segment neutrophil (%)	1.01 (0.98–1.04)	0.584		
CRP	1.01 (0.94–1.08)	0.886		

^†^reference condition: male.

Abbreviations: BMI, body mass index; CI, confidence interval; CRP, C-reactive protein; HR, hazard ratio; RCD, right colonic diverticulitis; WBC, white blood cell.

## Discussion

The results of our study comparing patients with RCD and patients with LCD show that the patients with RCD at our institution were younger and had milder diverticulitis severities and that the patients with LCD were more likely to experience recurrent diverticulitis.

Manabe *et al*.^12^ analysed data from 1,112 patients and found that LCD occurred more frequently in older patients than RCD did. However, their study was not designed to compare LCD and RCD. Chung *et al*.^9^ conducted a study that compared RCD and LCD but enrolled a small number of patients (n = 202). Although this previous study investigated diverticulitis severities, it did not identify cases of recurrent diverticulitis over a follow-up period. Recently, Soh *et al*.^10^ compared RCD with LCD and showed that patients with RCD were younger, had less advanced Hinchey stages, and had a lower recurrence rate (3.1% versus 17.9%). However, their study was limited by the enrolment of only 99 patients. An important strength of our study is that we enrolled 725 patients, which is a sufficiently large sample size for comparisons of RCD and LCD.

A recently published systematic review concerning acute colonic diverticulitis reported that younger ages are an important risk factor for recurrent diverticulitis^13^. In our study, as in previous studies, younger patients were more likely to experience recurrent diverticulitis (P = 0.026), although this association was not present in our subgroup analysis of patients with RCD (P = 0.075). This observation suggests that younger ages are associated with greater recurrence risks regardless of the diverticulitis location.

Ha *et al*.^14^ analysed data from 152 patients with RCD and reported finding no significant predictors of recurrence. However, Kim *et al*.^15^ analysed data from 296 patients and found that smoking (P = 0.011) and longer hospital stays (P < 0.001) predicted recurrence. However, these 2 studies omitted time as a variable in their analyses because they aimed to identify predictors of recurrence through a simple comparison of patients who did and did not experience recurrent diverticulitis. In our study, we identified predictors of recurrence with a Cox proportional regression analysis that included time as a variable. Smoking was not a significant predictor of recurrence (P = 0.111), but given the findings reported by Kim *et al*.^15^, we suspect that smoking may be relevant.

In our study, diabetes (P = 0.002) and hypertension (P < 0.001) were more common in patients with LCD than in those with RCD. This is probably attributable to older patients being more likely to have LCD. The patients with RCD had shorter average hospital stays (P < 0.001), which is consistent with the fact that they had less advanced modified Hinchey stages. Moreover, the patients with LCD were more likely to undergo emergency surgery during their hospitalisations, and this resulted in longer hospital stays.

The main strength of this study is that only patients with CT-confirmed diverticulitis were included. The patients’ diagnoses are therefore more reliable than those of patients in other studies who were diagnosed based solely on ultrasonography findings or clinical features^12,16^. Furthermore, our sample size was large enough to permit comparisons of the LCD and RCD groups.

One limitation of our study is that the overall recurrence rate of 7.8% was probably an undercount because we relied on a retrospective review of electric medical records. However, this recurrence rate is consistent with the 8.7% recurrence rate reported by a larger cohort study of 181,115 patients with colonic diverticulitis^17^. Another limitation is the relatively small number of patients with LCD included in our study. However, the LCD group was large enough to allow a meaningful comparison of LCD and RCD in a predominantly Asian population, for which a relatively small number of LCD cases would be expected. A third limitation is that we relied on data from a single centre.

## Conclusion

Relative to LCD, RCD occurs at younger ages, is associated with less advanced modified Hinchey stages, and is less likely to recur. Clinicians should therefore develop treatment strategies and follow-up protocols for RCD that are distinct from those for LCD. Although the recurrence rate for patients with RCD was lower than that for patients with LCD, future studies should focus on strategies for preventing recurrent RCD because RCD occurs at earlier ages than LCD does. Population-based studies with larger sample sizes are needed to check the accuracy of our findings.

## References

[1] Heise CP (2008). Epidemiology and pathogenesis of diverticular disease. J. Gastrointest. Surg..

[2] Painter NS, Burkitt DP (1971). Diverticular disease of the colon: a deficiency disease of Western civilization. Br. Med. J..

[3] Nakaji S (2002). Comparison of etiology of right-sided diverticula in Japan with that of left-sided diverticula in the West. Int. J. Colorectal Dis..

[4] Nakada I (1995). Diverticular disease of the colon at a regional general hospital in Japan. Dis. Colon. Rectum.

[5] Miura S (2000). Recent trends in diverticulosis of the right colon in Japan: retrospective review in a regional hospital. Dis. Colon. Rectum.

[6] Strate LL, Morris AM (2019). Epidemiology, Pathophysiology, and Treatment of Diverticulitis. Gastroenterology.

[7] Oh HK (2014). Surgical management of colonic diverticular disease: discrepancy between right- and left-sided diseases. World J. Gastroenterol..

[8] *The ASCRS textbook of colon and rectal surgery*. (Springer Science+Business Media 2016).

[9] Chung BH, Ha GW, Lee MR, Kim JH (2016). Management of Colonic Diverticulitis Tailored to Location and Severity: Comparison of the Right and the Left Colon. Ann. Coloproctol..

[10] Soh NYT (2018). Perforated diverticulitis: is the right and left difference present here too?. Int. J. Colorectal Dis..

[11] Ambrosetti P, Jenny A, Becker C, Terrier TF, Morel P (2000). Acute left colonic diverticulitis–compared performance of computed tomography and water-soluble contrast enema: prospective evaluation of 420 patients. Dis. Colon. Rectum.

[12] Manabe N (2015). Characteristics of Colonic Diverticulitis and Factors Associated With Complications: A Japanese Multicenter, Retrospective, Cross-Sectional Study. Dis. Colon. Rectum.

[13] Hupfeld L, Burcharth J, Pommergaard HC, Rosenberg J (2017). Risk factors for recurrence after acute colonic diverticulitis: a systematic review. Int. J. Colorectal Dis..

[14] Ha GW, Lee MR, Kim JH (2017). Efficacy of conservative management in patients with right colonic diverticulitis. Anz. J. Surg..

[15] Kim Yeong-Chan, Chung Jun-Won, Baek Jeong-Heum, Lee Won-Suk, Kim Doojin, Park Yeon-Ho, Yang Jun-Young, Lee Woon-Kee (2018). Risk Factors for Recurrence of Right Colonic Diverticulitis. Digestive Surgery.

[16] Mizuki A (2017). Changes in the Clinical Features and Long-term Outcomes of Colonic Diverticulitis in Japanese Patients. Intern. Med..

[17] Ho VP, Nash GM, Milsom JW, Lee SW (2015). Identification of diverticulitis patients at high risk for recurrence and poor outcomes. J. Trauma. Acute Care.

